# Aliens on Boats? The Eastern and Western Expansion of the African House Gecko

**DOI:** 10.3390/genes14020381

**Published:** 2023-01-31

**Authors:** Catarina Jesus Pinho, Luís Cardoso, Sara Rocha, Raquel Vasconcelos

**Affiliations:** 1 CIBIO, Centro de Investigação em Biodiversidade e Recursos Genéticos, InBIO Laboratório Associado, Campus de Vairão, Universidade do Porto, 4485-661 Vairão, Portugal; 2 Departamento de Biologia, Faculdade de Ciências da Universidade do Porto, 4169-007 Porto, Portugal; 3 BIOPOLIS Program in Genomics, Biodiversity and Land Planning, CIBIO, Campus de Vairão, 4485-661 Vairão, Portugal; 4 Phylogenomics Lab, Universidade de Vigo, 36310 Vigo, Spain

**Keywords:** invasive species, dispersal, distribution, *Hemidactylus mabouia*, *Hemidactlylus mercatorius*, Cabo Verde, Western Indian Ocean islands, mtDNA

## Abstract

Invasive species disrupt relations between endemics and their ecosystem and are an increasing biodiversity conservation problem. The *Hemidactylus* genus comprises the most successful invasive reptile species, including the worldwide-distributed *Hemidactylus mabouia*. In this study, we used 12S and ND2 sequences to taxonomically identify and tentatively determine the diversity and origin of these invaders in Cabo Verde while also clarifying this for several Western Indian Ocean (WIO) populations. By comparing our sequences to recently published ones, we showed, for the first time, that Cabo Verde individuals belong to the *H. mabouia sensu stricto* lineage and that both of its sublineages (a and b) occur there. Both haplotypes are also in Madeira, which indicates a connection between these archipelagos, possibly related to the past Portuguese trading routes. Across the WIO, results clarified the identity of many island and coastal populations, showing that this likely invasive *H. mabouia* lineage is widespread in the region, including northern Madagascar, with important conservation implications. Colonisation origins were difficult to access due to the wide geographical spread of these haplotypes; thus, several possible scenarios were outlined. The introduction of this species throughout western and eastern Africa may threaten endemic taxa and needs to be closely monitored.

## 1. Introduction

Since the industrial revolution, the displacement of native species by invasive species and their impact on the introduced ecosystems has been a problem for biodiversity conservation [1]. Hasty anthropogenic globalisation is leading geographically distant areas to become closer than they have ever been. The establishment of trade routes allowed an easy and faster way for species to relocate and enabled the establishment of populations in ecosystems that were otherwise unreachable [2]. Invasive species have been found to disrupt relations between endemic species and their ecosystem, for instance, by competition for resources, predation, and transmission of diseases [3,4]. These invaders can present several attributes that provide them with an advantage over native taxa, such as the possibility of repeated transport or the absence of natural predators and parasites [5]. Native (often endemic) species are at a disadvantage by not being adapted to compete with the invader. In this regard, reptiles are among the most affected groups since they are widely introduced, both unintentionally and through illegal trade, while there is a lack of studies and models to predict such invasions [6]. This problem increases in importance in insular ecosystems, which are often species-poor and disharmonic (i.e., with a biased taxa representation), although richer in endemic species than continents [7]. On islands, native species have often evolved and adapted to peculiar lifestyles, for example, to have smaller clutches with larger-sized offspring [7]. Additionally, insular populations tend to lose their escape behaviour due to the rarity or lack of predators and, thus, are more susceptible to introduced species [8]. Their usual small ranges make them particularly sensitive to anthropogenic changes and extinction [9].

The *Hemidactylus* genus comprises some of the most successful invasive reptile species in the world [10]. This fact is likely explained by their adaptations to travel great distances, such as fat reserves and calcareous and adhesive eggshells resistant to seawater [11]. The ability of some *Hemidactylus* species to thrive near humans has led to the displacement of endemic geckos in some biodiversity hotspots [12]. One example is *Hemidactylus mabouia* (Moreau de Jonnès, 1818), which currently has a worldwide distribution [13]. This species can easily establish populations in urban and semi-urban habitats by taking advantage of insect clusters that form around artificial light sources [14], inhibiting native species from the possibility of approaching these high food-density places [15,16]. This, combined with aggressive males that create a competitive environment surrounding these food sources, can lead to the displacement of native populations [17]. The morphology of this species, accompanied by its role in the ecosystem, can also be key factors in explaining its success. Combining ambush and pursuit tactics [18], *H. mabouia* has a limb proportion that may give it an advantage over native species [19].

A recent work suggests that *H. mabouia* originated in the Zambezian biogeographic region (Central–East Africa) and includes as many as 20 putative species-level lineages [10]. Of those, *H. mabouia sensu stricto* (native to the Congo Basin, West Africa; herein, *H. mabouia ss*) is invasive throughout Africa and the Americas [10]. According to that study, which focused on the variation of the latter across Africa and parts of the West Indies, Florida, and South America, two shallow subclades were detected. Both subclades arrived in the New World, presumably with the slave trade and recent global trade, likely through multiple colonisations [10]. Further translocations within the western hemisphere may have contributed to its spread, but additional sampling and higher-resolution molecular data are still needed to unveil this more deeply. However, the clear delimitation between *Hemidactylus mercatorius* Gray, 1882 and *H. mabouia* and the identity and origin of some Western Indian Ocean (WIO) islands and both eastern and western African populations remain particularly confusing, leading authors to refer to an ‘*H. mercatorius–mabouia* species complex’ and to suggest that *H. mercatorius* should be the designation applied to the Gulf of Guinea Islands, Malagasy, Comoran, and Seychelles populations pending further studies [20]. This led to the designation of the Cabo Verde populations also as *H. mercatorius* in some recent studies [21]. For simplicity, here we use the designation *H. mabouia*, currently used by the majority of the researchers to refer to populations outside Madagascar (e.g., [10]).

Due to increased globalisation, the number of translocated species has been rising, leading to more introduction events [22]. In fact, it was just found that Madeira Island also harbours both *H. mabouia ss* subclades’ most frequent haplotypes, which could have resulted from multiple introductions as well [23]. However, the number and origin of colonisations that explain that pattern remain uncertain with current data. These two subclades are often found in sympatry both in Africa and in South America; thus, multiple or a single colonisation event from a diverse population are plausible alternatives [23].

The presence of *H. mabouia* in Cabo Verde has been known since 2001 [24]. However, no thorough analysis has been performed so far concerning their genetic variation and putative origins. The Cabo Verde Archipelago is situated about 500 km off the west coast of Africa and comprises ten volcanic islands and several islets. It belongs to the biogeographical region of Macaronesia and the Mediterranean Basin biodiversity hotspot. It holds 32 endemic terrestrial reptile taxa from three genera, including four native species of the genus *Hemidactylus* [21,25], in addition to the introduced *Hemidactylus angulatus* Hallowell, 1854 and *H. mabouia*. The latter was first recorded on the island of São Vicente [24] and then on Santo Antão, Brava, and all nearby ports and main villages [21].

Across several WIO islands and eastern African coastal localities, the systematics and distribution of *Hemidactylus* are still particularly unclear. Based on previous works [20] that used different markers from the currently most comprehensive study [10], the Aldabra Archipelago (at the northwest of Madagascar) harbours a set of endemic haplotypes closely related to the most widespread Malagasy ones, assigned to *H. mercatorius*. However, the Comoros Archipelago (Mayotte), Seychelles (Mahé), Mascarenes (Reunion), several eastern African coastal localities throughout Tanzania (including Zanzibar and Pemba islands), and South Africa harbour haplotypes closely related to the ones assigned to a divergent lineage of *H. mercatorius* from North Madagascar [26,27]. Individuals from Uganda and the West African Gulf of Guinea islands, such as S. Tomé, Príncipe, and Annobón, also belong to this latter clade, which is hypothesised to reflect human-aided dispersals [10,28].

Taking advantage of the most recent data [10], the main goal of this work was to place these islands’ populations in this new detailed phylogenetic context for this species-complex, finding their closest phylogenetic affinities, as well as characterise their diversity and identify the possible geographical origin(s) of *H. mabouia* in Cabo Verde using molecular tools. Importantly, we compared them to populations from several West African and WIO locations, intending to definitely clarify their species–identity (*H. mabouia* vs. *H. mercatorius*). By sequencing several new samples from Cabo Verde and re-sequencing several representatives of the main haplogroups previously identified across the WIO and comparing them to the recently available phylogenetic data [10], we aim to answer the following questions. Are the introduced populations of *H. mabouia* in Cabo Verde derived from single or multiple events? What is the genetic variation of the species throughout the archipelago? To which of the 20 species-level lineages previously described do they belong? Where do the previously described *H. mercatorius–mabouia* lineages fall within the current phylogenetic framework for this group? With this work, we have clarified the taxonomic identity and origins of several island populations from both the western and eastern sides of the African continent.

## 2. Materials and Methods

### 2.1. Data Collection

**Figure 1:**
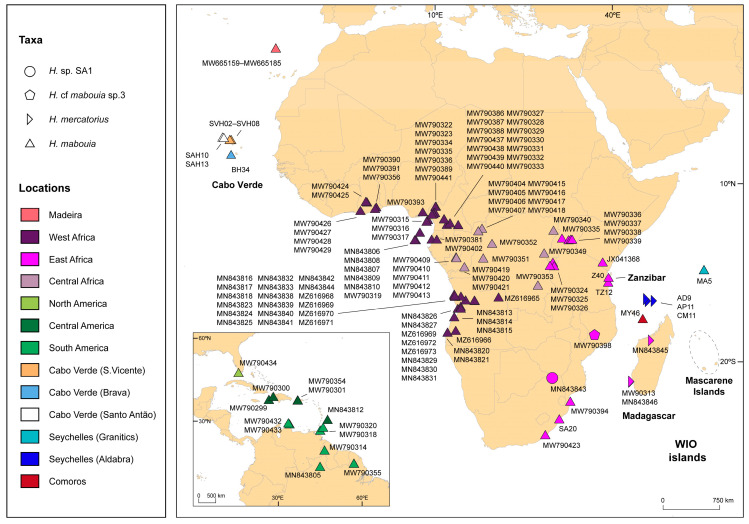
Map representing the geographical location of the samples used in this study for both 12S and ND2 markers. The bottom left corner represents samples from the Americas. Samples obtained from GenBank are represented by accession numbers, and samples obtained in this study are represented by tissue codes. Different symbols represent the different taxa and are coloured according to location. For further details, please check Table S1.

For Cabo Verde, we collected a total of 11 individuals in the field morphologically assigned to *H. mabouia* from 2006 to 2012. We collected and preserved their tail tips in 96% ethanol and successfully amplified and sequenced the mtDNA 12S ribosomal RNA marker for ten individuals. We further sequenced the mitochondrial gene ND2 in two of those specimens. To further clarify their taxonomy, we re-sequenced representatives of the main *H. mercatorius–mabouia* complex haplogroups previously identified in the WIO region for the ND2 marker [20] to enable data to be directly compared to other studies [10]. We sequenced five individuals from clade A-I, representing five different 16S haplotypes and localities (H1/MY46; H3/TZ12; H5/SA20; H7/MA5; H8/Z40; check [10]), plus three individuals from clade III (endemic to Aldabra Archipelago; check [10]), also from different islands (see details in Table S1 in Supplementary Material). All samples included in these analyses with available geographical coordinates are shown in Figure 1.

We extracted DNA from tissue samples using a standard high-salt protocol [29]. We amplified the 12S marker following a previous study [30]. We amplified the ND2 fragment using the primers MetF1/L4437 and H5934 [31]. For the PCR, we used an initial activation temperature of 95 °C for 15 min, a denaturation temperature of 95 °C for 30 s, annealing at 60 °C for 35 s, extension at 72 °C for 45 s for 35 cycles, and finally, 10 min at 72 °C.

### 2.2. Alignment and Phylogenetic Inference

For ND2, from a first visual examination of a larger alignment including representatives of the different species-level lineages identified in the previous study [10], it was clear that all our individuals were most similar to those identified as *H. mabouia ss* and *H. mercatorius* (only the ones from Aldabra Archipelago). Thus, for further analyses, we used only the most similar ND2 sequences to ours, 892 base pairs plus a couple of outgroup sequences (see below). For 12S, we collected from GenBank all available *Hemidactylus* 12S sequences that aligned with our fragment of 349 base pairs. In total, we retrieved 63 12S and 133 ND2 sequences and added them to our dataset. Detailed information about the locality and GenBank accession codes is presented in Table S1. We visualised all 12S and ND2 sequences using Geneious [32] and aligned them using MAFFT v7.505 [33] with the -auto option, which automatically selects the appropriate alignment strategy according to the data characteristics.

In detail, first, we built phylogenetic trees for the ND2 dataset with our 10 sequences, plus 127 *H. mabouia* ss sequences, three *H. mercatorius*, and one *Hemidactylus* c.f. *mabouia* sp. 3 plus two *Hemidactylus* sp. SA1 as outgroups. All sequences but ours are from the most recent and complete study on this group [10] (see details in Table S1 in Supplementary Material). These were collapsed into 46 haplotypes using ALTER [34]—only different haplotypes were kept except for the individuals sampled in this study. We used the IQ-TREE 2 [35] to infer a Maximum-Likelihood (ML) tree with 1000 bootstrap replicates. The best model (inferred through ModelFinder [36]) for the ML analyses was the TIM2 + F + G4. Then we used MrBayes 3.2 [37] to construct a phylogenetic tree under Bayesian inference (BI) using the same model. We ran BI analyses twice for 10^7^ generations and a sampling frequency of 10^3^ to produce 10^4^ trees. After examining the loglikelihood curves, we discarded 20% of the initial samples as burn-in. We considered ≥70% bootstrap proportion and ≥0.95 posterior probability as evidence for highly supported branches. For *H. mabouia ss*, as divergence was low, we further built a haplotype network with the 134 ND2 sequences to best visualise the relationships between all individuals using TCS [38] with the connection limit set to 40 steps, so that all haplotypes were connected as in the previous reference study [10]. Finally, we also constructed a haplotype network with 72 sequences for the 12S fragment using TCS with default parameters. We used FigTree v1.4.4 (https://github.com/rambaut/figtree, accessed on 12 July 2022) and tcsBU v1.21a [39] to visualise the results.

## 3. Results

**Figure 2:**
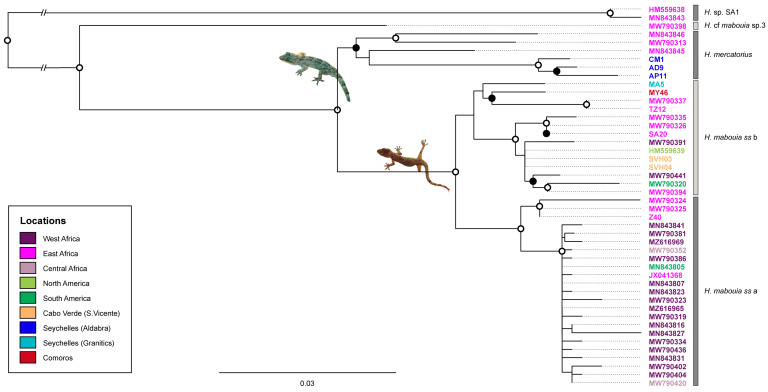
Maximum likelihood (ML) tree for the ND2 fragment showing the relationships between *Hemidactylus* from Cabo Verde and WIO region and their relatives. Accession numbers are colour coded geographically (for details, see Table S1). The designation of *H. mabouia* ss a/b follows a recent study [10]. Bootstrap (≥70%) and posterior probability (BI) support (>95%) are represented at nodes (solid black dots if there was ML support and open white dots if there was both ML and BI support). The two basal branches were shortened for graphical purposes, and *Hemidactylus* sp. SA1 was used as an outgroup [10]. See Figure 1 and Table S1 for detailed locality data and GenBank accession numbers of the new sequences.

ML and BI phylogenetic trees for the ND2 fragment had largely congruent topologies. The ML tree is shown in Figure 2 with support values from both methods. Both trees with detailed support values can be found in the Supplementary Materials (Figure S1). As in the previous study [10], they showed a clear distinction between the two major clades, *H. mercatorius* and *H. mabouia ss*, which were well-supported clades by both methods. Then two groups were present within *H. mabouia ss*: a highly supported one corresponding to *H. mabouia* ss a [10] and the other corresponding to *H. mabouia ss* b (unsupported; 52% bootstrap at ML). The two individuals from Cabo Verde sequenced for this marker (SVH03 and SVH04) are both well-nested into the *H. mabouia* ss b clade, exhibiting identical haplotypes. The WIO individuals fell in different clades: the ones from Aldabra Archipelago clearly grouped within *H. mercatorius* samples from Madagascar, most closely related to the sample from north-western Madagascar, while all the others (from Seychelles, Comoros, Tanzania, and South Africa) fell both within *H. mabouia* ss a and b.

**Figure 3:**
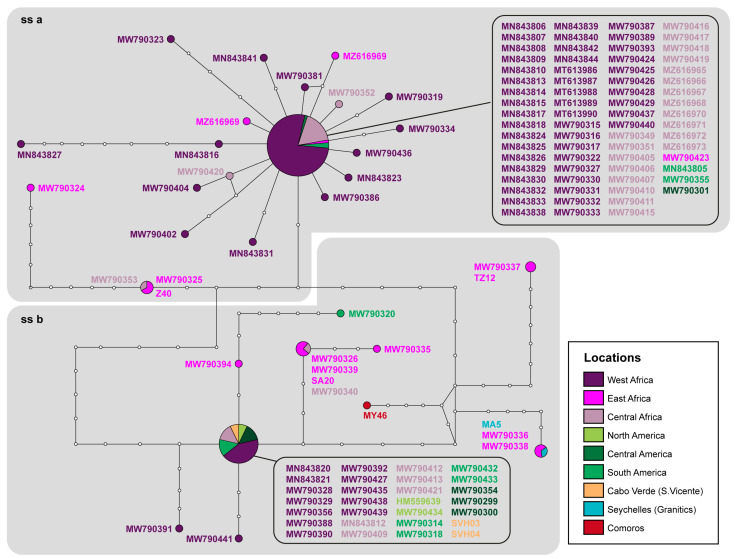
Haplotype network for ND2 mitochondrial marker. White dots along the branches represent missing or unsampled haplotypes. Haplotypes are colour coded geographically (for details, see Table S1) as well as the accession numbers/tissue codes which are given next to the nodes or in boxes. The circle size represents the haplotype frequency. The grey boxes in the network delimit *H. mabouia* ss a (**ss a**) and *H. mabouia* ss b (**ss b**), following the ML tree and a previous study [10].

**Figure 4:**
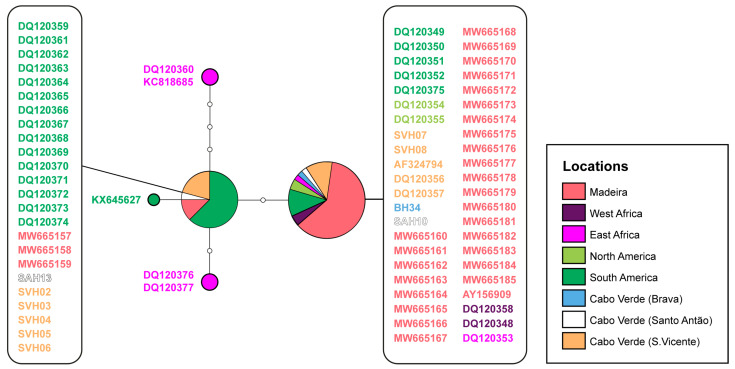
Haplotype network for 12S ribosomal RNA marker. White dots along the branches represent missing or unsampled haplotypes. Haplotypes are colour coded geographically (for details, see Table S1) as well as the accession numbers/tissue codes which are given next to the nodes or in boxes. The circle size represents the haplotype frequency.

The ND2 network (Figure 3) represents the relationships within the two *H. mabouia ss* clades in greater detail. The two major haplotypes widespread across central and western Africa reaching the Americas are clearly identifiable as the most frequent (and roughly central) of each clade. For this marker, only one haplotype was detected in Cabo Verde, precisely the most frequent one of *H. mabouia* ss clade b, in individuals that also shared the same 12S haplotype (Figure 4). For the individuals from the WIO, the situation is slightly different: several haplotypes were detected, belonging to both clades, but none corresponded to the most frequent and widespread of each clade. The sample from Zanzibar that clustered within *H. mabouia* ss a (Z40) exhibited a haplotype also found in Burundi and the Democratic Republic of Congo (DRC), a few mutation steps from the central one. The remaining samples fell within clade ss b. The South African sample (SA20) shared its haplotype with samples from Burundi, Uganda, and DRC; the Seychelles sample (MA5, from Mahé Island) exhibited a haplotype also found in Uganda, which is also the case of the sample from Tanzania (TZ12). A sample from Comoros Archipelago (MY46, from Mayotte Island) presents a unique haplotype, more or less equally distant from the latter two.

We were able to amplify and sequence a larger number of samples from Cabo Verde for 12S than for ND2, and thus a 12S haplotype network is represented in Figure 4. It further shows the presence also in Cabo Verde of the two major *H. mabouia* ss haplotypes, the same ones spread across the large areas of West Africa and the Americas, as well as in Madeira.

## 4. Discussion

Molecular phylogenetic methods are of great utility to precisely identify cases of cryptic species and lineages and tentatively infer colonisation or invasion routes, as is the case here. The close relationship between *H. mabouia ss* and *H. mercatorius* and the unclear identity of some populations was clearly shown in a previous study [20], to the point that some refer to specimens from Cabo Verde as *H. mercatorius* [21]. While these doubts remained for years due to the use of different gene fragments by different authors—16S rRNA was used [20], leading to incomparable datasets until now—the most recent study showed clearly that *H. mabouia* is a species-complex of circa 20 species-level lineages, as well as that the populations consensually called *H. mercatorius* (from central-south Madagascar) make *H. mabouia* paraphyletic [10]. Importantly, amongst the several *H. mabouia* lineages, they define *H. mabouia ss* as a sister taxon to *H. mercatorius* and a well-supported clade of two sublineages (a and b) widely distributed across sub-Saharan Africa, the West African islands of S. Tomé, Príncipe, and Bioko, and several localities of the Americas. Phylogeographic patterns and biogeographic reconstructions support the origin of *H. mabouia ss* in the East African Valley region, with relatively recent expansions throughout its western range, including to the Gulf of Guinea Islands and South America. At least throughout this western range, this species is most likely invasive, given its clear preference for human-dominated habitats and its proven ability to outcompete and directly prey on both native and other invasive geckos [14,16,17,40].

This new phylogenetic framework provided the ideal opportunity to further clarify the identity of several island and coastal populations on both sides of the African continent, including the ones from Cabo Verde, while at the same time exploring possible colonisation routes and origins (native or introduced) of several island populations, which are of utmost importance for conservation management. As such, the phylogenies now generated by combining representatives of the haplogroups/clades described in previous works [20] with the recently generated ND2 data provided us with important insights: populations from Cabo Verde are clearly assigned to *H. mabouia ss*, as well as all the representatives of the WIO populations, except for the ones of the Aldabra Archipelago, which are undoubtedly a lineage of *H. mercatorius* endemic to that archipelago. This allows us to say that the lineage referred to as *H. mabouia–mercatorius* clade A–I [20] are, in fact, *H. mabouia ss*, implying that these populations found in the islands of Mayotte (Comoros), Mahé (Seychelles), Reunion (Mascarenes) [41], Zanzibar, and Pemba (Tanzania), as well as the ones present in some (mostly coastal but not only) localities of both Tanzania and South Africa, are highly likely introduced through human-aided transport, which seems to be the predominant pattern of this species. This was, in fact, expected given their apparently restricted distribution and only recent detection across some of these locations (1905 and 2010 for Mahé and Reunion, respectively). With respect to the mainland locations where this species may have spread naturally vs. due to human aid, however, a much more detailed sampling throughout East Africa and higher resolution molecular data will be needed to properly clarify it. Populations from the Gulf of Guinea Island of Annobón (southwest of S. Tomé) [20] also belong to this clade. This is important information in terms of invasive species management, especially for those small islands where these introduced species may have a significant negative impact on native ones. Notably, these new results also allow us to infer that *H. mabouia ss* is present in northern localities of Madagascar (at least in Montagne des Français, Sambava, and Andapa—haplotypes H9, H11 and H37 from clade A–I [20]), corresponding to what has been erroneously interpreted as an *H. mercatorious* divergent lineage from that area [20,26,27]. Again, this adds to the list of invasive reptiles of these biodiversity hotspot islands and certainly has important conservation implications.

Regarding the genetic diversity of the introduced populations across these places, for Cabo Verde, it is now clear, taking together the 12S and ND2 results, that, similarly to what was found for Madeira and South America, both *H. mabouia ss* subclades are present, with individuals from subclade a (the most abundant one also in Madeira) present in S. Vicente, Santo Antão, and Brava and further populations assigned to subclade b in S. Vicente and Santo Antão for now. For ND2, only two individuals from Cabo Verde were successfully amplified (our samples were very old and most of them were too degraded to sequence), these exhibiting the most frequent and widespread haplotype from subclade b. However, for 12S, individuals from different islands were successfully amplified and sequenced, and one haplotype of each clade was found overall, and these were the same ones widespread across Madeira, South America, and also present in S. Tomé, Príncipe, and Bioko islands, clearly pointing to the expected scenario for this species of a relatively recent human-aided invasion of Cabo Verde. The fact that both haplotypes present in Cabo Verde are also present in Madeira indicates a possible connection between these archipelagos [23]. In fact, other studies have shown a possible colonisation route linking Madeira and Cabo Verde mediated by humans in mice *Mus musculus* [42]. This opposes previous studies, which claimed that *H. mabouia* found in Cabo Verde and Madeira all belong to one lineage [43] and that one of the haplotypes found in Madeira was exclusively related to South American individuals [23], and highlights the importance of a comprehensive sampling before any conclusions about dispersal patterns can be made, especially in species with cosmopolitan distributions, such as this one. Colonisation origins of the populations from Cabo Verde are still impossible to resolve due to the wide geographical spread of these haplotypes. Several scenarios are possible according to our results: a single south American origin from a place where both clades exist in sympatry, such as Brazil, as proposed for Madeira [23] or Puerto Rico [10]; a single colonisation from West Africa, again from a place where both clades co-habit; multiple colonisations both from South America or West Africa (or both); or a (single or double) colonisation from Madeira, such as hypothesised for *M. musculus* [42]. Further data with much higher resolution and a much more comprehensive sampling will be needed to distinguish these hypotheses and further clarify the phylogeographic and invasion patterns of this species.

In the WIO islands, the pattern was different. Although both *H. mabouia ss* subclades were again found in the region, none of the haplotypes found was the most frequent (and widespread) of each clade. On the contrary, there, we found low-frequency haplotypes some mutations away from the most frequent one, in most cases shared with East African individuals from Burundi, Cameroon, and Uganda from a previous study [10]. The sequenced representatives of *H. mercatorius–mabouia* clade A–I cover all the variation previously described for 16S within this group [20], which is completely unstructured, with many haplotypes shared between East African, Comoroan, and Seychelles populations. Only five representatives were sequenced in this work. The 16S data in a previous work [20] provided a more complete picture of the extensive degree of haplotype sharing across these locations. Altogether, this pattern most likely reflects multiple origins along the east part of the East African Rift Valley for these WIO islands populations, from a much more diverse genetic pool that the one on the western African coast. However, precise origins for these introductions of the island populations and clarification of the native vs. introduced origin of continental East African populations must await a much more comprehensive sampling across its East African distribution and, again, higher resolution markers. Given the case that haplotypes are most often identical to other locations, there would be no power in the current data to estimate colonisation ages. For that, a population genetics approach with higher resolution markers should follow.

Importantly, these inferences are so far based on mtDNA only. For a thorough look into dispersal and differentiation patterns and processes and species delimitations across this complex of circa 20 species-level lineages, multiple nuclear markers should be taken into account. These may also be important to understand if any genetic impact was caused in native closely related populations due to hybridisation and/or introgression. However, that was outside the scope of this work.

The introduction of invasive species to both Cabo Verde and the WIO islands can threaten endemic taxa. Since *H. mabouia* is one of the most effective invaders of the *Hemidactylus* genus [10], they should be closely monitored to ensure that they do not outcompete the native species. This is especially important, knowing that the other introduced *Hemidactylus* species in Cabo Verde, *H. angulatus*, has been displacing the native *Hemidactylus boavistensis* (Boulenger 1906) [44]. Additionally, in the WIO Mauritius Island (Mascarenes), food competition was demonstrated between the introduced *Hemidactylus frenatus* and the native diurnal gecko *Phelsuma ornata* [45], as well as its effect in displacing the nocturnal endemic *Nactus* species [12]. Overall, throughout the world, invasive *Hemidactylus* have been constantly associated with the decline in native species, especially other geckos, both through site competition and direct predation (e.g., [46,47,48]) For this reason, and especially now knowing their specific identity and its usual invasive behaviour, efforts should be made to clearly assess their current distribution and abundance across these different islands, and avoid their spread within and across them. Considering the noticeable fauna of endemic diurnal and nocturnal geckos across all these islands, their impact is expected to be considerable.

## 5. Conclusions

In summary, we clarify the identity of populations from Cabo Verde as *H. mabouia ss*, describe their current genetic variation across the archipelago, confirm the distinctiveness of the Aldabra Archipelago population as an endemic lineage of *H. mercatorius*, and assign other populations from the WIO islands, including populations from the north of Madagascar also to *H. mabouia ss*. Although precise colonisation sources could not be inferred due to the widespread distribution of identical haplotypes, this preliminary data shows that Cabo Verde is related to West African, Madeira, and South American populations, while WIO island populations have clear affinities with East African populations. Detailed sampling, particularly in East Africa, and higher resolution molecular data are still needed to infer with more detail the biogeographic history and spread of this species.

## Data Availability

The sequences generated in this article are available in the GenBank database (OQ266923-OQ266932 and OQ267597-OQ267606 accession codes).

## Supplementary Materials

The following supporting information can be downloaded at: https://www.mdpi.com/article/10.3390/genes14020381/s1, Table S1: Details of the *Hemidactylus* samples used in this study. The taxon (Hm SA1, *H.* sp. SA1; Hm sp3, *H.* cf. *mabouia* sp. 3; Hr, *H. mercatorius*; Hm, *H. mabouia sensu stricto*), tissue code, GenBank accession number (GenBank), country of origin (STP, São Tomé and Príncipe; DRC, Democratic Republic of the Congo), locality, geographical coordinates, and colour group of the sequences used for the two markers in this study are given. The * stands for new samples from this study; Figure S1: Phylogenetic trees. (A) Maximum likelihood tree, and (B) Bayesian inference for the ND2 fragment showing the relationships between *Hemidactylus* from Cabo Verde and WIO region and their relatives; File S1: Portuguese version of the abstract.

## References

[1] Paini D.R., Sheppard A.W., Cook D.C., De Barro P.J., Worner S.P., Thomas M.B. (2016). Global threat to agriculture from invasive species. Proc. Natl. Acad. Sci. USA.

[2] Ruiz G.M., Carlton J.T. (2003). Invasive species: Vectors and management strategies. Divers. Distrib..

[3] Gaiotto J.V., Abrahão C.R., Dias R.A., Bugoni L. (2020). Diet of invasive cats, rats and tegu lizards reveals impact over threatened species in a tropical island. Perspect. Ecol. Conserv..

[4] Willson J.D. (2017). Indirect effects of invasive Burmese pythons on ecosystems in southern Florida. J. Appl. Ecol..

[5] Alpert P. (2006). The advantages and disadvantages of being introduced. Biol. Invasions.

[6] Reed R., Kraus F. (2010). Invasive reptiles and amphibians: Global perspectives and local solutions. Anim. Conserv..

[7] Novosolov M., Raia P., Meiri S. (2013). The island syndrome in lizards. Glob. Ecol. Biogeogr..

[8] Cooper W.E., Pyron R.A., Garland T. (2014). Island tameness: Living on islands reduces flight initiation distance. Proc. R. Soc. B Boil. Sci..

[9] Soares N., Gonçalves J.F., Vasconcelos R., Ribeiro R.P. (2022). Combining multiple data sources to predict IUCN conservation status of reptiles. Advances in Intelligent Data Analysis XX, Proceedings of the 20th International Symposium on Intelligent Data Analysis, IDA 2022, Rennes, France, 20–22 April 2022.

[10] Agarwal I., Ceríaco L.M., Metallinou M., Jackman T.R., Bauer A.M. (2021). How the African house gecko (*Hemidactylus mabouia*) conquered the world. R. Soc. Open Sci..

[11] Bansal R., Karanth K.P. (2013). Phylogenetic analysis and molecular dating suggest that *Hemidactylus anamallensis* is not a member of the *Hemidactylus* radiation and has an ancient late Cretaceous origin. PLoS ONE.

[12] Cole N.C., Jones C.G., Harris S. (2005). The need for enemy-free space: The impact of an invasive gecko on island endemics. Biol. Conserv..

[13] Weterings R., Vetter K.C. (2018). Invasive house geckos (*Hemidactylus* spp.): Their current, potential and future distribution. Curr. Zool..

[14] Hughes D.F., Meshaka W.E., van Buurt G. (2015). The superior colonizing gecko *Hemidactylus mabouia* on Curaçao: Conservation implications for the native gecko *Phyllodactylus martini*. J. Herpetol.

[15] Buurt G.V. (2004). Field Guide to the Reptiles and Amphibians of Aruba, Curaçao and Bonaire.

[16] Williams R., Pernetta A.P., Horrocks J.A. (2016). Outcompeted by an invader? Interference and exploitative competition between tropical house gecko (*Hemidactylus mabouia*) and Barbados leaf-toed gecko (*Phyllodactylus pulcher*) for diurnal refuges in anthropogenic coastal habitats. Integr. Zool..

[17] Short K.H., Petren K. (2012). Rapid species displacement during the invasion of Florida by the tropical house gecko *Hemidactylus mabouia*. Biol. Invasions.

[18] Dornburg A., Lippi C., Federman S., Moore J.A., Warren D.L., Iglesias T.L., Brandley M.C., Watkins-Colwell G.J., Lamb A.D., Jones A. (2016). Disentangling the influence of urbanization and invasion on endemic geckos in tropical biodiversity hot spots: A case study of *Phyllodactylus martini* (Squamata: Phyllodactylidae) along an urban gradient in Curaçao. Bull. Peabody Mus. Nat. Hist..

[19] Zaaf A., Van Damme R. (2001). Limb proportions in climbing and ground-dwelling geckos (Lepidosauria, Gekkonidae): A phylogenetically informed analysis. Zoomorphology.

[20] Rocha S., Carretero M.A., Harris D.J. (2010). On the diversity, colonization patterns and status of *Hemidactylus* spp. (Reptilia: Gekkonidae) from the Western Indian Ocean islands. Herpetol. J..

[21] Vasconcelos R., Brito J.C., Carranza S., Harris D.J. (2013). Review of the distribution and conservation status of the terrestrial reptiles of the Cape Verde Islands. Oryx.

[22] Simberloff D. (2013). Invasive Species: What Everyone Needs to Know.

[23] Rato C., Martins B., Rocha R., Silva-Rocha I. (2021). Uncovered genetic diversity in *Hemidactylus mabouia* (Reptilia: Gekkonidae) from Madeira Island reveals uncertain sources of introduction. Amphibia-Reptilia.

[24] Jesus J., Brehm A., Pinheiro M., Harris D.J. (2001). Relationships of *Hemidactylus* (Reptilia: Gekkonidae) from the Cape Verde Islands: What mitochondrial DNA data indicate. J. Herpetol..

[25] Koehler G., Vasconcelos R., Köhler G., Geniez P., Crochet P.-A. (2020). A new endemic species of *Hemidactylus* (Squamata: Gekkonidae) from São Nicolau Island, Cabo Verde. Zootaxa.

[26] Vences M., Wanke S., Vieites D.R., Branch W.R., Glaw F., Meyer A. (2004). Natural colonization or introduction? Phylogeographical relationships and morphological differentiation of house geckos (*Hemidactylus*) from Madagascar. Biol. J. Linn. Soc..

[27] Boumans L., Vieites D.R., Glaw F., Vences M. (2007). Geographical patterns of deep mitochondrial differentiation in widespread Malagasy reptiles. Mol. Phylogenetics Evol..

[28] Rocha S., Carretero M.A., Harris D.J. (2005). Diversity and phylogenetic relationships of *Hemidactylus* geckos from the Comoro islands. Mol. Phylogenetics Evol..

[29] Sambrook J., Fritsch E.F., Maniatis T. (1989). Molecular Cloning: A Laboratory Manual.

[30] Arnold E., Vasconcelos R., Harris D., Mateo J., Carranza S. (2008). Systematics, biogeography and evolution of the endemic *Hemidactylus* geckos (Reptilia, Squamata, Gekkonidae) of the Cape Verde Islands: Based on morphology and mitochondrial and nuclear DNA sequences. Zool. Scr..

[31] Macey J.R., Larson A., Ananjeva N.B., Fang Z., Papenfuss T.J. (1997). Two novel gene orders and the role of light-strand replication in rearrangement of the vertebrate mitochondrial genome. Mol. Biol. Evol..

[32] Kearse M., Moir R., Wilson A., Stones-Havas S., Cheung M., Sturrock S., Buxton S., Cooper A., Markowitz S., Duran C. (2012). Geneious Basic: An integrated and extendable desktop software platform for the organisation and analysis of sequence data. Bioinformatics.

[33] Katoh K., Standley D.M. (2013). MAFFT multiple sequence alignment software version 7: Improvements in performance and usability. Mol. Biol. Evol..

[34] Glez-Peña D., Gomez-Blanco D., Reboiro-Jato M., Fdez-Riverola F., Posada D. (2010). ALTER: Program-oriented conversion of DNA and protein alignments. Nucleic Acids Res..

[35] Minh B.Q., Schmidt H.A., Chernomor O., Schrempf D., Woodhams M.D., von Haeseler A., Lanfear R. (2020). IQ-TREE 2: New models and efficient methods for phylogenetic inference in the genomic era. Mol. Biol. Evol..

[36] Kalyaanamoorthy S., Minh B.Q., Wong T.K.F., von Haeseler A., Jermiin L.S. (2017). ModelFinder: Fast model selection for accurate phylogenetic estimates. Nat. Methods.

[37] Ronquist F., Teslenko M., Van Der Mark P., Ayres D.L., Darling A., Höhna S., Larget B., Liu L., Suchard M.A., Huelsenbeck J.P. (2012). MrBayes 3.2: Efficient Bayesian phylogenetic inference and model choice across a large model space. Syst. Biol..

[38] Crandall M.C.D.P.K., Clement M., Posada D. (2000). TCS: A computer program to estimate gene genealogies. Mol. Ecol..

[39] Murias dos Santos A., Cabezas M.P., Tavares A.I., Xavier R., Branco M. (2016). tcsBU: A tool to extend TCS network layout and visualisation. Bioinformatics.

[40] Du Tertre J.-B. (1654). Histoire Générale des Isles de S. Christophe, de la Guadeloupe, de la Martinique et Autres dans l’Amérique.

[41] Sanchez M., Rocha S., Probst J.-M. (2012). Un nouveau gecko nocturne naturalisé sur l’île de La Réunion: *Hemidactylus mercatorius* Gray, 1842 (Reptilia: Squamata: Gekkonidae). Bull. Soc. Herp. Fr..

[42] Santos A.S.C.d. (2017). História da Colonização do Ratinho-Caseiro, *Mus musculus domesticus*, em Ilhas Atlânticas (Madeira, Açores e Cabo Verde): Uma Abordagem Multilocus. Ph.D. Thesis.

[43] Jesus J., Brehm A., Harris D.J. (2005). Phylogenetic relationships of *Hemidactylus* geckos from the Gulf of Guinea islands: Patterns of natural colonisations and anthropogenic introductions estimated from mitochondrial and nuclear DNA sequences. Mol. Phylogenetics Evol..

[44] López-Jurado L.F., Mateo J.A., Geniez P. (1999). Los reptiles de la Isla de Boavista (archipiélago de Cabo Verde). Bol. Asoc. Herpetol. Esp..

[45] Cole N. (2005). The new noisy neighbours: Impacts of alien house geckos on endemics in Mauritius. Aliens-Invasive Species Spec. Group Newsl..

[46] Fuenmayor G.R., Ugueto G., Bauer A.M., Barros T., Manzanilla J. (2005). Expansion and natural history of a successful colonising gecko in Venezuela (Reptilia: Gekkonidae: *Hemidactylus mabouia*) and the discovery of *H. frenatus* in Venezuela. Herpetol. Rev..

[47] Rödder D., Solé M., Böhme W. (2008). Predicting the potential distribution of two alien invasive Housegeckos (Gekkonidae: *Hemidactylus frenatus*, *Hemidactylus mabouia*). North-West. J. Zool..

[48] van Buur G. (2006). Conservation of amphibians and reptiles in Aruba, Curaçao and Bonaire. Appl. Herpetol..

