# Supplementary material for: Aliens on Boats? The Eastern and Western Expansion of the African House Gecko
Source: Genes (Basel). 2023 Jan 31;14(2):381. doi: 10.3390/genes14020381 (PMC9957147; doi:10.3390/genes14020381)
Supplement: Supplementary file 1 [file genes-14-00381-s001.zip › genes-2164640-File S1.pdf]

Abstract in Portuguese

Espécies invasoras perturbam as relações entre espécies endémicas e respectivos ecossistemas e são um problema crescente de conservação da biodiversidade. O género *Hemidactylus* apresenta as espécies de répteis invasores de maior sucesso, como o *H. mabouia* distribuído globalmente. Neste estudo, usámos sequências de 12S e ND2 para identificar taxonomicamente e tentar determinar a diversidade e origem desses invasores em Cabo Verde, enquanto esclarecemos também isso para várias populações do Oceano Índico Ocidental (OIO). Comparando as nossas sequências com as recentemente publicadas, mostrámos, pela primeira vez, que os indivíduos de Cabo Verde pertencem à linhagem *H. mabouia sensu stricto* e que as duas sublinhagens desta (a e b) ocorrem aí. Ambos os haplótipos também se encontram na Madeira, o que indica uma possível ligação entre estes arquipélagos, possivelmente relacionada com as antigas rotas comerciais portuguesas. Em todo o OIO, os resultados esclareceram a identidade de muitas populações insulares e costeiras, mostrando que esta linhagem provavelmente invasora de *H. mabouia* está disseminada na região, incluindo no Norte de Madagáscar, com importantes implicações de conservação. As origens da colonização foram difíceis de definir devido à ampla distribuição geográfica desses haplótipos, assim vários cenários possíveis foram delineados. A introdução desta espécie na África Ocidental e Oriental pode ameaçar taxa endémicos e precisa ser cuidadosamente monitorizada.
