# Supplementary material for: Aliens on Boats? The Eastern and Western Expansion of the African House Gecko
Source: Genes (Basel). 2023 Jan 31;14(2):381. doi: 10.3390/genes14020381 (PMC9957147; doi:10.3390/genes14020381)
Supplement: Supplementary file 1 [file genes-14-00381-s001.zip › genes-2164640-supplementary-Table S1.pdf]

| Taxon | Voucher code | GeneBank | Marker | Country | Locality | Latitude | Longitude | Group |
|---|---|---|---|---|---|---|---|---|
| *Hm SA1* | CAS 248776 | MN843843 | ND2 | South Africa | Limpopo Prov. | -22.7064 | 29.8289 | East Africa |
| *Hm SA1* | AMB 8301 | HM559638 | ND2 | South Africa | Limpopo Prov. | - | - | East Africa |
| *Hm sp3* | MVZ 265935 | MW790398 | ND2 | Mozambique | Zambezia | -15.4639 | 36.9778 | East Africa |
| *Hr* | AMNH R-16825 | MN843845 | ND2 | Madagascar | Mahajanga | -16.3833 | 47.0381 | East Africa |
| *Hr* | AMNH-R-165402 | MW790313 | ND2 | Madagascar | Toliara | -23.3511 | 43.6870 | East Africa |
| *Hr* | AMNH R-16826 | MN843846 | ND2 | Madagascar | Toliara | -23.3006 | 43.6592 | East Africa |
| *Hr* | AP11* | OQ266924 | ND2 | Seychelles | Assumption, Aldabra | -9.7342 | 46.5003 | Seychelles (Aldabra) |
| *Hr* | AD9* | OQ266923 | ND2 | Seychelles | Picard, Aldabra | -9.4011 | 46.2060 | Seychelles (Aldabra) |
| *Hr* | CM1* | OQ266925 | ND2 | Seychelles | Menai Island, Aldabra | -9.7100 | 47.5079 | Seychelles (Aldabra) |
| *Hm* | YPM 14798 | HM559639 | ND2 | USA | Florida | - | - | North America |
| *Hm* | LSUMZ 12403 | MN843805 | ND2 | Brazil | Roraima | 1.7958 | -61.5281 | South America |
| *Hm* | MB03-000965 | MN843806 | ND2 | STP | Príncipe | 1.6257 | 7.4138 | West Africa |
| *Hm* | MB03-000998 | MN843807 | ND2 | STP | São Tomé | 0.3382 | 6.7323 | West Africa |
| *Hm* | MB03-001025 | MN843808 | ND2 | STP | Príncipe | 1.6259 | 7.4138 | West Africa |
| *Hm* | MB03-001059 | MN843809 | ND2 | STP | São Tomé | 0.3499 | 6.5411 | West Africa |
| *Hm* | MB03-001060 | MN843810 | ND2 | STP | São Tomé | 0.3499 | 6.5411 | West Africa |
| *Hm* | XXXX 267704 | MN843812 | ND2 | Barbados | St. Joseph | 13.1988 | -59.5403 | Central America |
| *Hm* | UF 187193 | MN843813 | ND2 | Angola | Benguela | -12.6245 | 13.2327 | West Africa |
| *Hm* | CAS 263381 | MN843814 | ND2 | Angola | Benguela | -12.6245 | 13.2327 | West Africa |
| *Hm* | CAS 263382 | MN843815 | ND2 | Angola | Benguela | -12.6245 | 13.2327 | West Africa |
| *Hm* | CAS 263373 | MN843816 | ND2 | Angola | Luanda | -9.1830 | 13.3706 | West Africa |
| *Hm* | CAS 263374 | MN843817 | ND2 | Angola | Luanda | -9.1830 | 13.3706 | West Africa |
| *Hm* | CAS 263375 | MN843818 | ND2 | Angola | Luanda | -9.1830 | 13.3706 | West Africa |
| *Hm* | CAS 263522 | MN843820 | ND2 | Angola | Namibe | -15.2089 | 12.1015 | West Africa |
| *Hm* | CAS 263523 | MN843821 | ND2 | Angola | Namibe | -15.2089 | 12.1015 | West Africa |
| *Hm* | CAS 263518 | MN843823 | ND2 | Angola | Luanda | -8.9977 | 13.2638 | West Africa |
| *Hm* | INBAC/AMB 1066 | MN843824 | ND2 | Angola | Luanda | -8.8155 | 13.2359 | West Africa |
| *Hm* | MHNC-UP/Rep 494 | MN843825 | ND2 | Angola | Malanje | -9.7712 | 15.1572 | West Africa |
| *Hm* | CAS 263432 | MN843826 | ND2 | Angola | Kwanza Sul | -11.1981 | 13.8355 | West Africa |
| *Hm* | CAS 263433 | MN843827 | ND2 | Angola | Kwanza Sul | -11.1981 | 13.8355 | West Africa |
| *Hm* | CAS 263431 | MN843829 | ND2 | Angola | Kwanza Sul | -10.8675 | 14.3216 | West Africa |
| *Hm* | CAS 263430 | MN843830 | ND2 | Angola | Kwanza Sul | -10.8672 | 14.3244 | West Africa |
| *Hm* | UF 187201 | MN843831 | ND2 | Angola | Kwanza Sul | -10.8675 | 14.3228 | West Africa |
| *Hm* | CAS 258427 | MN843832 | ND2 | Angola | Malanje | -9.8727 | 16.7009 | West Africa |
| *Hm* | INBAC/JVV 9516 | MN843833 | ND2 | Angola | Luanda | -9.1839 | 13.3722 | West Africa |
| *Hm* | INBAC/JVV 9524 | MN843838 | ND2 | Angola | Luanda | -9.1839 | 13.3722 | West Africa |
| *Hm* | INBAC/JVV 9525 | MN843839 | ND2 | Angola | Luanda | -9.1839 | 13.3722 | West Africa |
| *Hm* | INBAC/JVV 9529 | MN843840 | ND2 | Angola | Luanda | -9.1839 | 13.3722 | West Africa |
| *Hm* | INBAC/JVV 9531 | MN843841 | ND2 | Angola | Luanda | -9.1839 | 13.3722 | West Africa |
| *Hm* | INBAC/JVV 9535 | MN843842 | ND2 | Angola | Luanda | -9.1839 | 13.3722 | West Africa |
| *Hm* | CAS 263443 | MN843844 | ND2 | Angola | Luanda | -9.1839 | 13.3722 | West Africa |
| *Hm* | PEM R19461 | MZ616965 | ND2 | Angola | Lunda | -8.4184 | 20.7419 | West Africa |
| *Hm* | NB1083 | MZ616966 | ND2 | Angola | Huíla | -14.9189 | 13.4853 | West Africa |
| *Hm* | FKH0443 | MZ616967 | ND2 | Angola | Cuanza | -9.1431 | 14.3672 | West Africa |
| *Hm* | FKH0444 | MZ616968 | ND2 | Angola | Cuanza | -9.1431 | 14.3672 | West Africa |
| *Hm* | FKH0031 | MZ616969 | ND2 | Angola | Cuanza | -11.1972 | 14.3406 | West Africa |
| *Hm* | FKH0219 | MZ616970 | ND2 | Angola | Luanda | -9.0606 | 13.3539 | West Africa |
| *Hm* | FKH0288 | MZ616971 | ND2 | Angola | Malanje | -9.8194 | 16.6539 | West Africa |
| *Hm* | P9-220 | MZ616972 | ND2 | Angola | Cuanza | -11.1972 | 14.3406 | West Africa |

| | | | | | | | | |
|---|---|---|---|---|---|---|---|---|
| *Hm* | P9-221 | MZ616973 | ND2 | Angola | Cuanza | -11.1972 | 14.3406 | West Africa |
| *Hm* | MB03-001025 | MT613986 | ND2 | STP | Príncipe | - | - | West Africa |
| *Hm* | MB03-001060 | MT613987 | ND2 | STP | São Tomé | - | - | West Africa |
| *Hm* | MB03-001059 | MT613988 | ND2 | STP | São Tomé | - | - | West Africa |
| *Hm* | MB03-000965 | MT613989 | ND2 | STP | Príncipe | - | - | West Africa |
| *Hm* | MB03-000998 | MT613990 | ND2 | STP | Príncipe | - | - | West Africa |
| *Hm* | 269034 ND2 | MW790299 | ND2 | Haiti | Sud | 18.1090 | -73.9403 | Central America |
| *Hm* | 269269 ND2 | MW790300 | ND2 | Haiti | Gonave Island | 18.8349 | -72.8666 | Central America |
| *Hm* | AMB 4151 | MW790301 | ND2 | Puerto Rico | Guánica | 17.9535 | -66.8485 | Central America |
| *Hm* | BPN 1215 | MW790314 | ND2 | Guyana | Imbaimadai | 5.6925 | -60.2820 | South America |
| *Hm* | CAS 207964 | MW790315 | ND2 | Equatorial Guinea | Bioko Norte | 3.7518 | 8.7834 | West Africa |
| *Hm* | CAS 207968 | MW790316 | ND2 | Equatorial Guinea | Bioko Norte | 3.7518 | 8.7834 | West Africa |
| *Hm* | CAS 207984 | MW790317 | ND2 | Equatorial Guinea | Bioko Norte | 3.4613 | 8.5524 | West Africa |
| *Hm* | CAS 231714 | MW790318 | ND2 | Trinidad & Tobago | Tunapuna-Piarco | 10.5926 | -61.3474 | South America |
| *Hm* | CAS 233427 | MW790319 | ND2 | STP | Príncipe | 1.6399 | 7.4193 | West Africa |
| *Hm* | CAS 245322 | MW790320 | ND2 | Trinidad & Tobago | Tobago | 11.2928 | -60.6289 | South America |
| *Hm* | CAS 249873 | MW790322 | ND2 | Cameroon | Northwest | 6.0097 | 10.1295 | West Africa |
| *Hm* | CAS 249883 | MW790323 | ND2 | Cameroon | Northwest | 5.9661 | 10.0420 | West Africa |
| *Hm* | CAS 250633 | MW790324 | ND2 | Burundi | Gitega | -3.4271 | 29.9346 | East Africa |
| *Hm* | CAS 250796 | MW790325 | ND2 | Burundi | Rutana | -4.0277 | 30.1507 | East Africa |
| *Hm* | CAS 250810 | MW790326 | ND2 | Burundi | Bururi | -3.9796 | 29.4369 | East Africa |
| *Hm* | CAS 253263 | MW790327 | ND2 | Cameroon | Central | 3.8987 | 11.5122 | West Africa |
| *Hm* | CAS 253264 | MW790328 | ND2 | Cameroon | Central | 3.8987 | 11.5122 | West Africa |
| *Hm* | CAS 253316 | MW790329 | ND2 | Cameroon | East | 2.9007 | 13.9033 | West Africa |
| *Hm* | CAS 253317 | MW790330 | ND2 | Cameroon | East | 2.9007 | 13.9033 | West Africa |
| *Hm* | CAS 253460 | MW790331 | ND2 | Cameroon | South | 2.9399 | 11.9763 | West Africa |
| *Hm* | CAS 253461 | MW790332 | ND2 | Cameroon | South | 2.9399 | 11.9763 | West Africa |
| *Hm* | CAS 253610 | MW790333 | ND2 | Cameroon | South | 3.1983 | 12.5228 | West Africa |
| *Hm* | CAS 253849 | MW790334 | ND2 | Cameroon | Littoral | 4.9538 | 9.8661 | West Africa |
| *Hm* | CAS 255988 | MW790335 | ND2 | Uganda | Central | 0.6167 | 31.4749 | East Africa |
| *Hm* | CAS 256075 | MW790336 | ND2 | Uganda | Kampala District | 0.3413 | 32.5838 | East Africa |
| *Hm* | CAS 256079 | MW790337 | ND2 | Uganda | Central | 0.3996 | 33.0105 | East Africa |
| *Hm* | CAS 256086 | MW790338 | ND2 | Uganda | Mukono District | 0.4370 | 32.9543 | East Africa |
| *Hm* | CAS 256279 | MW790339 | ND2 | Uganda | Eastern | 0.4293 | 33.1959 | East Africa |
| *Hm* | CRSN84 | MW790340 | ND2 | DRC | Orientale | 1.9589 | 30.0357 | Central Africa |
| *Hm* | EBG 1287 | MW790349 | ND2 | DRC | South Kivu | -1.8747 | 28.4524 | Central Africa |
| *Hm* | ELI 1945 | MW790351 | ND2 | DRC | Bandundu | -2.7300 | 18.1443 | Central Africa |
| *Hm* | ELI 2274 | MW790352 | ND2 | DRC | Equateur | -0.2594 | 19.6358 | Central Africa |
| *Hm* | ELI 342 | MW790353 | ND2 | DRC | Katanga | -7.2936 | 27.3947 | Central Africa |
| *Hm* | JB 218 | MW790354 | ND2 | Puerto Rico | Guanica | 17.9705 | -66.9094 | Central America |
| *Hm* | LSUMZ 13920 | MW790355 | ND2 | Brazil | Para | 2.5027 | -53.0449 | South America |
| *Hm* | LSUMZ 20258 | MW790356 | ND2 | Ghana | Greater Accra Region | 5.8391 | 0.1085 | West Africa |
| *Hm* | MCZR-187693 | MW790381 | ND2 | Gabon | Estuaire | 0.4487 | 9.4124 | West Africa |
| *Hm* | MHNG 271551 | MW790386 | ND2 | Cameroon | Central | 3.8762 | 11.5119 | West Africa |
| *Hm* | MHNG 271552 | MW790387 | ND2 | Cameroon | Central | 3.8762 | 11.5119 | West Africa |
| *Hm* | MHNG 271553 | MW790388 | ND2 | Cameroon | Central | 3.8762 | 11.5119 | West Africa |
| *Hm* | MHNG 271554 | MW790389 | ND2 | Cameroon | Littoral | 4.7166 | 9.7277 | West Africa |
| *Hm* | MVZ 245309 | MW790390 | ND2 | Ghana | Greater Accra Region | 5.8803 | 0.0378 | West Africa |
| *Hm* | MVZ 249705 | MW790391 | ND2 | Ghana | Greater Accra Region | 5.6074 | -0.1718 | West Africa |
| *Hm* | MVZ 253216 | MW790392 | ND2 | Nigeria | Akwa Ibom | 5.0527 | 7.9039 | West Africa |

| | | | | | | | | |
|---|---|---|---|---|---|---|---|---|
| *Hm* | MVZ 253217 | MW790393 | ND2 | Nigeria | Akwa Ibom | 5.0527 | 7.9039 | West Africa |
| *Hm* | MVZ 265926 | MW790394 | ND2 | Mozambique | Maputo | -26.8478 | 32.8839 | East Africa |
| *Hm* | NCSM 76794 | MW790402 | ND2 | Gabon | Estuaire | 0.4536 | 10.2781 | West Africa |
| *Hm* | NMNH 576055 | MW790404 | ND2 | DRC | Likouala | 1.5142 | 17.9378 | West Africa |
| *Hm* | NMNH 576056 | MW790405 | ND2 | DRC | Likouala | 1.5142 | 17.9378 | Central Africa |
| *Hm* | NMNH 576114 | MW790406 | ND2 | DRC | Likouala | 1.0769 | 17.2997 | Central Africa |
| *Hm* | NMNH 576122 | MW790407 | ND2 | DRC | Likouala | 1.0769 | 17.2997 | Central Africa |
| *Hm* | NMNH 584223 | MW790409 | ND2 | DRC | Lekoumou | -2.5393 | 13.5427 | Central Africa |
| *Hm* | NMNH 584224 | MW790410 | ND2 | DRC | Lekoumou | -2.6668 | 13.5958 | Central Africa |
| *Hm* | NMNH 584225 | MW790411 | ND2 | DRC | Lekoumou | -2.6668 | 13.5958 | Central Africa |
| *Hm* | NMNH 584226 | MW790412 | ND2 | DRC | Lekoumou | -2.6752 | 13.5860 | Central Africa |
| *Hm* | NMNH 584227 | MW790413 | ND2 | DRC | Lekoumou | -2.6668 | 13.5958 | Central Africa |
| *Hm* | NMNH 584296 | MW790415 | ND2 | DRC | Likouala | 1.0881 | 17.3074 | Central Africa |
| *Hm* | NMNH 584302 | MW790416 | ND2 | DRC | Likouala | 1.0881 | 17.3074 | Central Africa |
| *Hm* | NMNH 584322 | MW790417 | ND2 | DRC | Likouala | 1.0881 | 17.3074 | Central Africa |
| *Hm* | NMNH 584323 | MW790418 | ND2 | DRC | Likouala | 1.0881 | 17.3074 | Central Africa |
| *Hm* | NMNH 584366 | MW790419 | ND2 | DRC | Pool | -4.1865 | 14.9570 | Central Africa |
| *Hm* | NMNH 584368 | MW790420 | ND2 | DRC | Pool | -4.1865 | 14.9570 | Central Africa |
| *Hm* | NMNH 584370 | MW790421 | ND2 | DRC | Pool | -4.1865 | 14.9570 | Central Africa |
| *Hm* | TM 84932 | MW790423 | ND2 | South Africa | Eastern Cape | -32.4777 | 28.6514 | East Africa |
| *Hm* | UWBM 6004 | MW790424 | ND2 | Ghana | Ashanti Region | 6.7366 | -1.5294 | West Africa |
| *Hm* | UWBM 6005 | MW790425 | ND2 | Ghana | Ashanti Region | 6.8297 | -1.7214 | West Africa |
| *Hm* | UWBM 9094 | MW790426 | ND2 | Ghana | Western Region | 5.2818 | -2.6417 | West Africa |
| *Hm* | UWBM 9095 | MW790427 | ND2 | Ghana | Western Region | 5.2818 | -2.6417 | West Africa |
| *Hm* | UWBM 9111 | MW790428 | ND2 | Ghana | Western Region | 5.2818 | -2.6417 | West Africa |
| *Hm* | UWBM 9112 | MW790429 | ND2 | Ghana | Western Region | 5.2818 | -2.6417 | West Africa |
| *Hm* | YPM 18123 | MW790432 | ND2 | Dutch Antilles | Curação | 12.1196 | -68.8805 | South America |
| *Hm* | YPM 18131 | MW790433 | ND2 | Dutch Antilles | Curação | 12.3754 | -69.1567 | South America |
| *Hm* | YPM 14798 | MW790434 | ND2 | USA | Florida | 24.6743 | -81.3879 | North America |
| *Hm* | ZFMK 75377 | MW790435 | ND2 | Cameroon | Littoral | 4.9172 | 9.9892 | West Africa |
| *Hm* | ZFMK 89590 | MW790436 | ND2 | Cameroon | Littoral | 4.7166 | 9.7277 | West Africa |
| *Hm* | ZFMK 89591 | MW790437 | ND2 | Cameroon | Central | 3.8675 | 11.5204 | West Africa |
| *Hm* | ZFMK 89592 | MW790438 | ND2 | Cameroon | Central | 3.8762 | 11.5119 | West Africa |
| *Hm* | ZFMK 89593 | MW790439 | ND2 | Cameroon | Central | 3.8762 | 11.5119 | West Africa |
| *Hm* | ZFMK 89594 | MW790440 | ND2 | Cameroon | Central | 3.8762 | 11.5119 | West Africa |
| *Hm* | ZFMK 89595 | MW790441 | ND2 | Cameroon | Southwest | 4.6326 | 9.4353 | West Africa |
| *Hm* | JEM1864 | JX041368 | ND2 | Kenya | Taita Taveta | -3.4000 | 38.3640 | East Africa |
| *Hm* | SA20* | OQ266928 | ND2 | South Africa | Durban | -29.8333 | 31.0167 | East Africa |
| *Hm* | TZ12* | OQ266931 | ND2 | Tanzania | Dar Es Salaam | -6.8050 | 39.2896 | East Africa |
| *Hm* | MA5* | OQ266926 | ND2 | Seychelles | Airport, Mahé | -4.6717 | 55.5108 | Seychelles (Granitics) |
| *Hm* | MY46* | OQ266927 | ND2 | Comoros | Chirongoui, Mayotte | -12.9360 | 45.1443 | Comoros |
| *Hm* | Z40* | OQ266932 | ND2 | Tanzania | Upenja, Zanzibar | -5.9942 | 39.3525 | East Africa |
| *Hm* | Madeira1 12S | AY156909 | 12S | Portugal | Madeira | - | - | Madeira |
| *Hm* | DB31558 | MW665160 | 12S | Portugal | Madeira | 32.6472 | −16.969386 | Madeira |
| *Hm* | DB31405 | MW665161 | 12S | Portugal | Madeira | 32.6443 | −16.915567 | Madeira |
| *Hm* | DB31536 | MW665162 | 12S | Portugal | Madeira | 32.6443 | −16.915567 | Madeira |
| *Hm* | DB31559 | MW665163 | 12S | Portugal | Madeira | 32.6470 | −16.973176 | Madeira |
| *Hm* | DB31560 | MW665164 | 12S | Portugal | Madeira | 32.6472 | −16.969386 | Madeira |
| *Hm* | DB31537 | MW665165 | 12S | Portugal | Madeira | 32.6443 | −16.915567 | Madeira |
| *Hm* | DB31432 | MW665166 | 12S | Portugal | Madeira | 32.6443 | −16.915567 | Madeira |

| | | | | | | | | |
|---|---|---|---|---|---|---|---|---|
| *Hm* | DB31552 | MW665167 | 12S | Portugal | Madeira | 32.6472 | −16.969386 | Madeira |
| *Hm* | DB31562 | MW665168 | 12S | Portugal | Madeira | 32.6472 | −16.969386 | Madeira |
| *Hm* | DB31524 | MW665169 | 12S | Portugal | Madeira | 32.6443 | −16.915567 | Madeira |
| *Hm* | DB31549 | MW665170 | 12S | Portugal | Madeira | 32.6443 | −16.915567 | Madeira |
| *Hm* | DB31554 | MW665171 | 12S | Portugal | Madeira | 32.6472 | −16.969386 | Madeira |
| *Hm* | DB31563 | MW665172 | 12S | Portugal | Madeira | 32.6472 | −16.969386 | Madeira |
| *Hm* | DB31525 | MW665173 | 12S | Portugal | Madeira | 32.6443 | −16.915567 | Madeira |
| *Hm* | DB31550 | MW665174 | 12S | Portugal | Madeira | 32.6443 | −16.915567 | Madeira |
| *Hm* | DB31555 | MW665175 | 12S | Portugal | Madeira | 32.6472 | −16.969386 | Madeira |
| *Hm* | DB31344 | MW665176 | 12S | Portugal | Madeira | 32.6443 | −16.915567 | Madeira |
| *Hm* | DB31526 | MW665177 | 12S | Portugal | Madeira | 32.6443 | −16.915567 | Madeira |
| *Hm* | DB31345 | MW665178 | 12S | Portugal | Madeira | 32.6443 | −16.915567 | Madeira |
| *Hm* | DB31527 | MW665179 | 12S | Portugal | Madeira | 32.6443 | −16.915567 | Madeira |
| *Hm* | DB31346 | MW665180 | 12S | Portugal | Madeira | 32.6443 | −16.915567 | Madeira |
| *Hm* | DB31533 | MW665181 | 12S | Portugal | Madeira | 32.6443 | −16.915567 | Madeira |
| *Hm* | DB31428 | MW665182 | 12S | Portugal | Madeira | 32.6443 | −16.915567 | Madeira |
| *Hm* | DB31561 | MW665183 | 12S | Portugal | Madeira | 32.6472 | −16.969386 | Madeira |
| *Hm* | DB31548 | MW665184 | 12S | Portugal | Madeira | 32.6443 | −16.915567 | Madeira |
| *Hm* | DB31557 | MW665185 | 12S | Portugal | Madeira | 32.6472 | −16.969386 | Madeira |
| *Hm* | DB31553 | MW665157 | 12S | Portugal | Madeira | 32.6470 | −16.973176 | Madeira |
| *Hm* | DB31564 | MW665158 | 12S | Portugal | Madeira | 32.6470 | −16.973176 | Madeira |
| *Hm* | DB31556 | MW665159 | 12S | Portugal | Madeira | 32.6472 | −16.969386 | Madeira |
| *Hm* | E609.14 | DQ120376 | 12S | Kenya | Kilifi District | - | - | East Africa |
| *Hm* | E609.20 | DQ120377 | 12S | Uganda | Masaka District | - | - | East Africa |
| *Hm* | E509.21 | DQ120360 | 12S | Kenya | Kilifi District | - | - | East Africa |
| *Hm* | JS173 | KC818685 | 12S | Uganda | - | - | - | East Africa |
| *Hm* | E609.21 | DQ120353 | 12S | Uganda | Rukungiri District | - | - | East Africa |
| *Hm* | E609.22 | DQ120354 | 12S | USA | Florida | - | - | North America |
| *Hm* | E609.23 | DQ120355 | 12S | USA | Florida | - | - | North America |
| *Hm* | Bichoa | AF324794 | 12S | Cabo Verde | S. Vicente | - | - | Cabo Verde (S. Vicente) |
| *Hm* | 31HbouSV | DQ120356 | 12S | Cabo Verde | S. Vicente | - | - | Cabo Verde (S. Vicente) |
| *Hm* | 32HbouSV | DQ120357 | 12S | Cabo Verde | S. Vicente | - | - | Cabo Verde (S. Vicente) |
| *Hm* | SVH02* | OQ267600 | 12S | Cabo Verde | S. Vicente | 16.8572 | -24.9811 | Cabo Verde (S. Vicente) |
| *Hm* | SVH03* | OQ267601<br>OQ266929 | 12S/<br>ND2 | Cabo Verde | S. Vicente | 16.8576 | -24.9797 | Cabo Verde (S. Vicente) |
| *Hm* | SVH04* | OQ267602<br>OQ266930 | 12S/<br>ND2 | Cabo Verde | S. Vicente | 16.8576 | -24.9797 | Cabo Verde (S. Vicente) |
| *Hm* | SVH05* | OQ267603 | 12S | Cabo Verde | S. Vicente | 16.8576 | -24.9797 | Cabo Verde (S. Vicente) |
| *Hm* | SVH06* | OQ267604 | 12S | Cabo Verde | S. Vicente | 16.8576 | -24.9797 | Cabo Verde (S. Vicente) |
| *Hm* | SVH07* | OQ267605 | 12S | Cabo Verde | S. Vicente | 16.8839 | -25.0269 | Cabo Verde (S. Vicente) |
| *Hm* | SVH08* | OQ267606 | 12S | Cabo Verde | S. Vicente | 16.8923 | -24.9895 | Cabo Verde (S. Vicente) |
| *Hm* | BH34* | OQ267597 | 12S | Cabo Verde | Brava | 14.8695 | -24.6955 | Cabo Verde (Brava) |
| *Hm* | SAH10* | OQ267598 | 12S | Cabo Verde | Santo Antão | 17.1819 | -25.0648 | Cabo Verde (Santo Antão) |
| *Hm* | SAH13* | OQ267599 | 12S | Cabo Verde | Santo Antão | 17.0214 | -25.3316 | Cabo Verde (Santo Antão) |
| *Hm* | E609.5 | DQ120349 | 12S | Brazil | Genipabu | - | - | South America |
| *Hm* | E609.7 | DQ120350 | 12S | Brazil | Genipabu | - | - | South America |
| *Hm* | E609.11 | DQ120351 | 12S | Brazil | Fernando de Noronha | - | - | South America |
| *Hm* | E609.12 | DQ120352 | 12S | Brazil | Fernando de Noronha | - | - | South America |
| *Hm* | E609.9 | DQ120375 | 12S | Brazil | Rio Grande do Norte | - | - | South America |
| *Hm* | E609.10 | DQ120359 | 12S | Brazil | Fernando de Noronha | - | - | South America |

| | | | | | | | | |
|---|---|---|---|---|---|---|---|---|
| *Hm* | E1109.8 | DQ120361 | 12S | Trinidad & Tobago | Tobago | - | - | South America |
| *Hm* | E1109.9 | DQ120362 | 12S | Trinidad & Tobago | Tobago | - | - | South America |
| *Hm* | E509.22 | DQ120363 | 12S | Argentina | Iguazu | - | - | South America |
| *Hm* | E609.1 | DQ120364 | 12S | Brazil | Mato Grosso | - | - | South America |
| *Hm* | E609.2 | DQ120365 | 12S | Brazil | Mato Grosso | - | - | South America |
| *Hm* | E609.3 | DQ120366 | 12S | Brazil | Mato Grosso | - | - | South America |
| *Hm* | E609.4 | DQ120367 | 12S | Brazil | Mato Grosso | - | - | South America |
| *Hm* | E609.6 | DQ120368 | 12S | Brazil | Rio Grande do Norte | - | - | South America |
| *Hm* | E609.8 | DQ120369 | 12S | Brazil | Rio Grande do Norte | - | - | South America |
| *Hm* | E609.13 | DQ120370 | 12S | Brazil | Pernambuco | - | - | South America |
| *Hm* | E609.18 | DQ120371 | 12S | Trinidad & Tobago | Trinidad | - | - | South America |
| *Hm* | E609.19 | DQ120372 | 12S | Trinidad & Tobago | Trinidad | - | - | South America |
| *Hm* | E609.24 | DQ120373 | 12S | Puerto Rico | Península de Jobos | - | - | South America |
| *Hm* | E1109.1 | DQ120374 | 12S | Puerto Rico | Península de Jobos | - | - | South America |
| *Hm* | CURC-R 113 | KX645627 | 12S | Uruguay | - | - | - | South America |
| *Hm* | E1708.13 | DQ120348 | 12S | Equatorial Guinea | Bioko Island | - | - | West Africa |
| *Hm* | 37HmabSTo | DQ120358 | 12S | STP | São Tomé | - | - | West Africa |
