# Supplementary figures and images for: Aliens on Boats? The Eastern and Western Expansion of the African House Gecko
Source: Genes (Basel). 2023 Jan 31;14(2):381. doi: 10.3390/genes14020381 (PMC9957147; doi:10.3390/genes14020381)

A

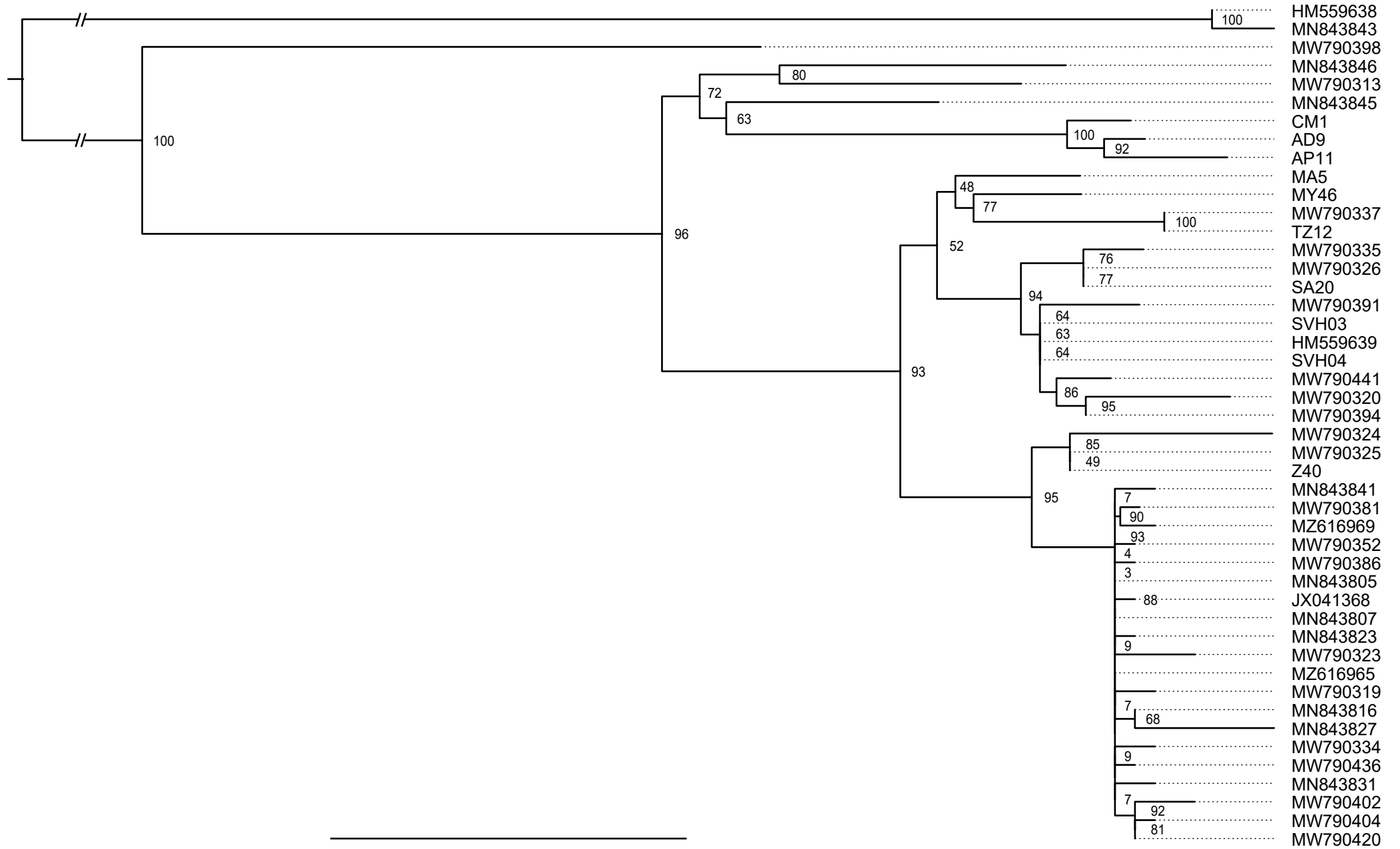

B

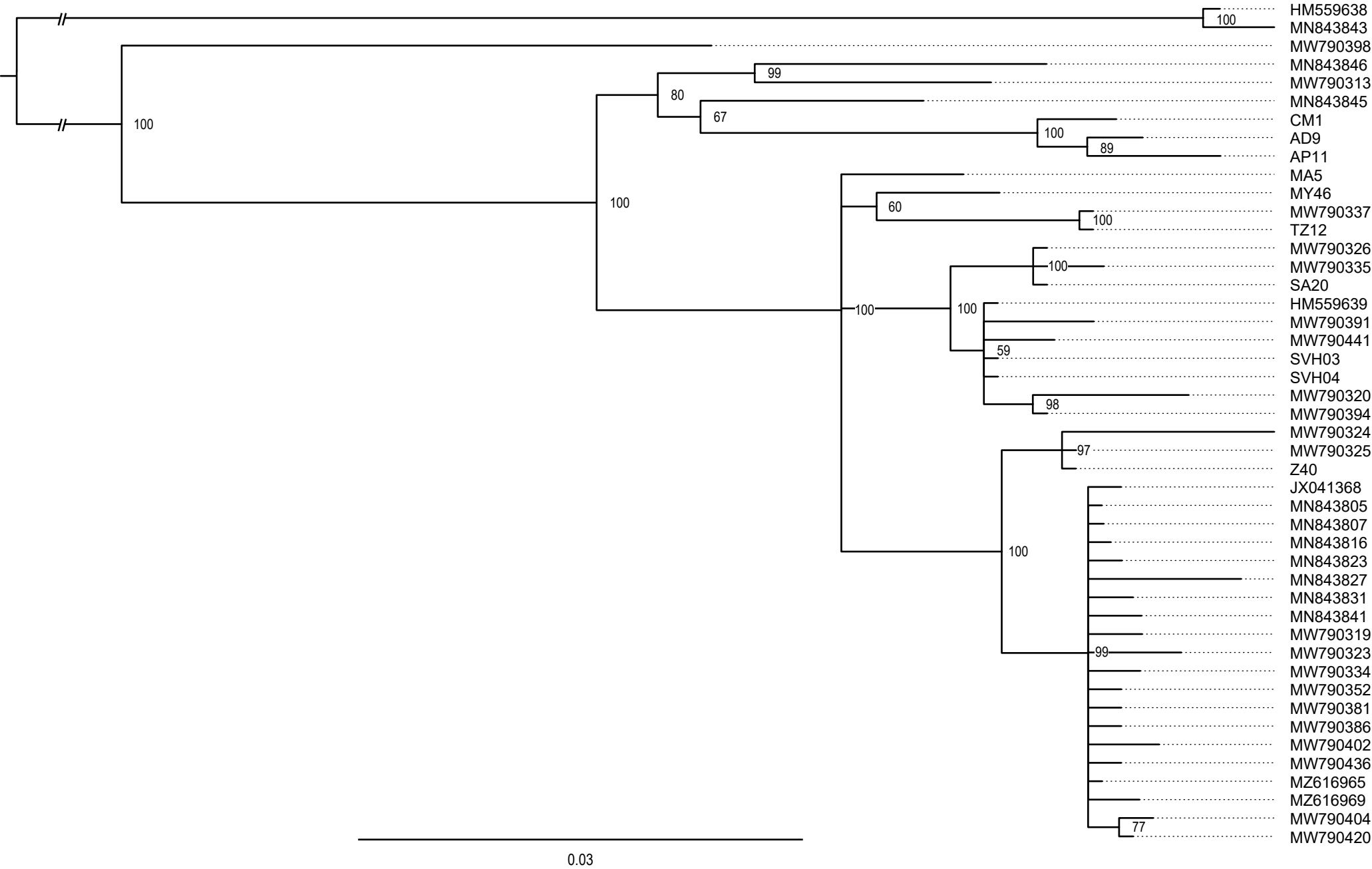

Supplement: Supplementary file 1 [file genes-14-00381-s001.zip › FigS1.pdf]
